# Microwave Irradiation of Nanohydroxyapatite from Chicken Eggshells and Duck Eggshells

**DOI:** 10.1155/2014/275984

**Published:** 2014-10-14

**Authors:** Nor Adzliana Sajahan, Wan Mohd Azhar Wan Ibrahim

**Affiliations:** Department of Biomedical Engineering, Faculty of Engineering, University of Malaya and PPP, 50603 Kuala Lumpur, Malaysia

## Abstract

Due to similarity in composition to the mineral component of bones and human hard tissues, hydroxyapatite with chemical formula Ca_10_(PO_4_)_6_(OH)_2_ has been widely used in medical field. Both chicken and duck eggshells are mainly composed of calcium carbonate. An attempt has been made to fabricate nanohydroxyapatite (nHA) by chicken (CES) and duck eggshells (DES) as calcium carbonate source (CaCO_3_). CES and DES were reacted with diammonium hydrogen [(NH_4_)_2_HPO_4_] solution and subjected to microwave heating at 15 mins. Under the effect of microwave irradiation, nHA was produced directly in the solution and involved in crystallographic transformation. Sample characterization was done using by X-ray diffraction (XRD), fourier transform infrared spectroscopy (FTIR), and scanning electron microscopy (SEM).

## 1. Introduction

Hard tissue is one of the crucial components of our body. Natural bone is a type of nanocomposite containing organic (30%) and inorganic compounds (70%) in a form of needle- or rod-like materials with length of 25–50 nm [1, 2]. There are many alternatives for the replacement of bone due to trauma, accident, or disease. These include autografts, allografts, xenografts, and synthetic biomaterials. However, these methods have some complications. For instance, autografts confront the risk of bone defect of donor-site, limited availability of grafts and it requires additional surgeries for harvesting [3]. Hydroxyapatite (HA) has been reliable as bone substitute due to chemically and biologically resemblance with human native tissue in addition to its crystallographic structure [4, 5]. Generally, HA (Ca_10_(PO_4_)_6_(OH)_2_) constitutes about 69% of the weight of human bone [6]. It is bioactive, that is, forms a strong bond with bone which enhances the implant fixation and provides stability to the interface between bone and HA ceramic. This characteristic has made HA being used in orthopedics field and in dental implants [7, 8]. This ceramic has been widely known for its properties such as excellent biocompatibility, nontoxicity, noninflammatory, and pyrogenetic response [8, 9]. HA has also been extensively used as bone filler, implant coating, and cell-culture carrier [10]. Due to poor mechanical reliability of HA ceramic, its applications are limited to nonloading bearing such as bone graft substitution and coatings on metallic implants [11]. It was reported that the debris of HA formed was deposited between the implant interface and the surrounding tissue which encouraged the irritation in cells such as monocytes or macrophages to release inflammatory mediators, cytokines, and matrix metalloproteinases, inducing cytotoxicity and pathologic bone resorption [12]. HA can be produced by various synthesis routes including chemical precipitation method [13], hydrothermal techniques [8], sol-gel route [14], and mechanochemical [15] techniques using different sources of calcium and phosphorus as starting materials. These methods, however, have several disadvantages. Most of HA powders prepared by chemical precipitation route have calcium deficiency and HA powders produced from these methods exhibit thermal decomposition of HA to *α*- or *β*-tricalcium phosphate phase [13]. The obtained HA powders from sol-gel method usually have poor crystallinity and thermal stability [16]. Hydrothermal process is said to be an expensive synthesis method since it involves several steps of heat treatment [17]. Zhang and Vecchio [18] have synthesized HA at temperature of 120°C to 180°C by using hydrothermal method. The precursors used were dicalcium phosphate anhydrous (CaHPO_4_) and calcium carbonate (CaCO_3_). At temperature of 120°C, the CaHPO_4_ was still found after being subjected to heating for 24 hr while HA was formed along with *β*-TCP above temperature of 140°C. The particles size of HA obtained was 200 nm in width and several microns in length. Compared with conventional method, microwave synthesis can be used to overcome these disadvantages as it offers fast reaction, easy reproducibility, narrow particle distribution, high yield, high purity, efficient energy transformation, and throughout volume heating [9]. During microwave heating, the sample itself becomes the source of heat. This is due to the fact that the heat is generated internally within the material instead of originating from external heating and subsequent radiative transfer [9, 19]. It also provides a rapid and shorter synthesis time as its high depths penetration enables the entire substance to be subjected to uniform and rapid heating [17, 20]. This causes the thermal gradients to minimize and reduce the time for particle diffusion; thus the product can be formed in a shorter time [20]. With the aid of soft template of cetyltrimethylammonium (CTAB), Arami et al. [21] have successfully synthesized nanostrips HA by microwave synthesis technique. The use of CTAB helps in epitaxial growth of nanostructures and controls the size and morphology of the HA. The size of HA obtained was 10 and 55 nm in width and length, respectively, with a very short time of microwave heating (2.45 GHz, 900 W) of 5 minutes. No secondary phases were found. In another work, Meejoo et al. [19] have performed a simple microwave-assisted wet precipitation of HA by immediately subjecting the mixture solution of the starting materials to microwave heating (850 W) for 20 min. This work did not require a pH control as reported by others. This is because the calcium hydroxide, Ca(OH)_2_ precursor, used is a strong base. Even though the HA obtained was calcium deficient HA, it was completely removed after annealing it at 800°C. A needle-shaped particle with nanosize of 50 nm in diameter and 200 nm in length has been achieved using this method. In this work, HA powder was synthesized from both chicken and duck eggshells as calcium source using microwave irradiation. This study proposed a straightforward, economic, and reproducible technique for fabrication of HA powder using various waste materials. The effect of microwave power as well as irradiation time on particles morphology will be observed. The characteristics of HA powder produced will be studied for phase composition using X-ray diffraction (XRD), powder morphology using scanning electron microscope (SEM), and functional groups using fourier transform infra-red (FTIR).

## 2. Materials and Methods

The commercial (NH_4_)_2_HPO_4_ and 25% liquor ammonia were of analytical grade and used without further purification. (NH_4_)_2_HPO_4_ and eggshells were used as phosphate and calcium precursor, respectively. CES were collected and repetitively washed with distilled water and their membranes were peeled off. They were further boiled in water for 15 min to remove dirt and organic residue. The cleaned and dried eggshells were then ground to fine powder using agate mortar and pestle. The procedure of this experiment was adapted from Nayar and Guha [22]. The experimental procedure has been modified by increasing the chemical reagent used so that it is suitable for microwave technique and to increase the yield of powder. 25 g of CES powder was dissolved in 100 mL 1 : 3 of hydrochloric acid, HCl, and distilled water. The mixture was stirred rigorously for 30 minutes with the help of magnetic stirrer. It was then filtered to eliminate the residue of eggshells. 1850 mL (NH_4_)_2_HPO_4_ solution was added to the eggshells solution and stirred thoroughly. The mixture pH was maintained 10-11 by adding dropwise of 1 : 1 of 25% liquor ammonia and distilled water. The mixture was immediately subjected to microwave heating in a domestic oven operating at 2.45 GHz (900 W) for 15 min. The precipitate formed was centrifuged at the rotation speed of 3000 rpm several times with distilled water to eliminate the remaining ammonia. It was then dried overnight at 100°C. As-dried precipitate was ground to fine powder. The experiment was repeated exactly using DES. This procedure instruction was repeated for several times obtaining identical phases of the nano-HA powder. The microwave power (700 W and 900 W) and irradiation time (15 min, 30 min and 45 min) were varied to study their effect on the HA particles morphology.  Table 1 shows the synthesis condition and sample ID. The powder samples were analyzed by XRD (Panalytical) using Cu-K*α* radiation (1.540600 Å) with voltage and current settings of 45 kV and 40 mA, respectively. The XRD patterns were recorded in step size (°2Th.) of 0.03° and step time 1 s. FTIR (Nicolet iS10) was used to determine the functional groups of the sample powders. The FTIR spectra were recorded in the region 400–4000 cm^−1^ using the KBr pellet technique. The morphology of sample powders was obtained with a Hitachi model S-3500N scanning electron microscope, operated at 20 kV with magnification of 100 kx. Small amount of powder was coated with gold palladium using a sputter coater. Then it was held at the sample holder to observe the images.

## 3. Result and Discussion

One of the important parameters that affects the complete reaction of HA is alkaline pH around 9-10 [10]. After the mixing of eggshells solution and (NH_4_)_2_HPO_4_, the preliminary pH of the mixture was around 7-8 pH. It was adjusted to 10-11 pH by using 25% liquor ammonia. It is found that above pH 10, pure crystalline HA phases with narrow size distribution and uniform morphology occurred [10]. However, pH lower than 10 contributes to the presence of *β*-TCP and the morphology of the HA particles tends to be irregular and flaky [10]. The addition of ammonium hydroxide is said to prevent the formation of carbonate during synthesis [23]. Palanivelu et al. [24] found out that, upon synthesized HA at neutral pH 7, secondary phases of calcium deficient HA such as octacalcium phosphate (OCP) and dicalcium phosphate (DCP) also occurred. This was due to the slow rate reaction of ion releasing between the calcium and phosphate precursors in the solution medium. In addition, it also caused the agglomeration of particles. Conversely, the fast rate of mobility of both ions above pH 9 led to agglomeration. However, it was found that ultrasonic irradiation techniques are suitable for producing HA at pH 9.

**Figure 1:**
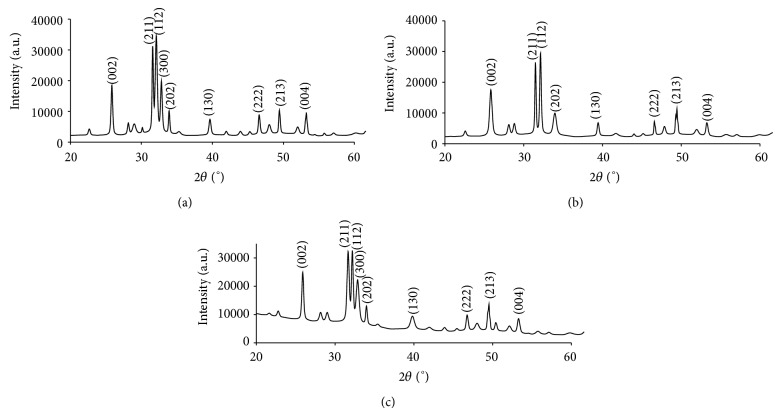
X-ray diffraction patterns of powder samples (a) dHA-900, (b) cHA-900, and (c) dHA-700.

The XRD pattern for product from duck eggshells and chicken eggshells powders is shown in Figure 1. The phase identification was done according to standard JCPDS file 00-024-0033. Phase analysis revealed that all major peaks of HA were presented in both powders except for peak at 2*θ* angle of 32.87° for cHA-900 sample and were in a good agreement with standard data for HA which confirmed the formation of HA during microwave heating. However, the disappearance of the peak was not known.

Since microwave radiation provides a rapid heating rate and uniform heating throughout the entire volume of the substance, the energy was sufficient to produce the major peaks of HA at planes of (002), (211), (112), (300), (202), (130), (222), (213), and (004). The XRD patterns of the as-synthesized powder show prominent peaks at 2*θ* angle of 25.82°, 31.57°, 32.07°, 32.79°, 34.00°, 39.63°, 46.56°, and 49.41° for dHA-A900 while cHA-900 shows high intensity peaks at 25.81°, 31.55°, 32.11°, 33.93°, 39.42°, 46.56°, and 49.37°. For dHA-700, the XRD peaks also appeared at 25.87°, 31.65°, 32.17°, 32.83°, 33.96°, 39.78°, 46.73°, and 49.54°. The absence of other peaks corresponding to impurities such as *β*-TCP, *α*-TCP, and calcium carbonate indicates that a pure phase of HA has been synthesized as seen in Figure 1. Upon varying the irradiation time of 15 min, 30 min, and 45 min (results for XRD and FTIR, i.e., 30 min and 45 min were not shown and it was done for DES only), it was found that from XRD analysis there was no indication of other secondary phases in all the samples. All peaks corresponding to HA were well presented. The efficient transformation of energy and heating of microwave synthesis caused well-crystallized HA powders to synthesize without calcification or sintering process or further heating as has always been reported by other studies. This can be seen at the sharp and high intensity peaks of both XRD patterns. Prabakaran et al. [25] have successfully synthesized a stoichiometric HA by wet chemical method. They used chicken eggshells as calcium source and the precipitate formed was calcined at various temperatures of 400°C, 700°C, and 900°C. It was observed that the calcination process did not alter the phase composition of the HA but the intensity of the XRD pattern became sharper as the temperature rose, that is, crystallinity of the HA tends to be well-crystallized.

**Figure 2:**
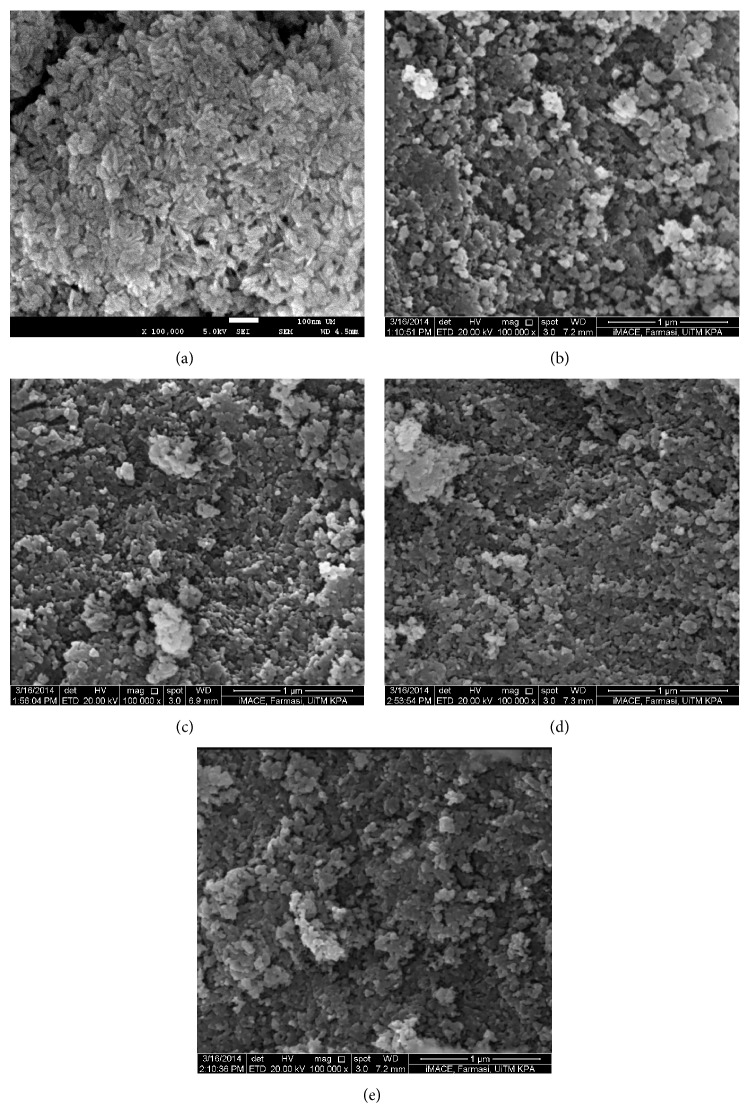
Scanning electron micrographs of (a) dHA-700, (b) cHA-900, (c) dHA-A900, (d) dHA-B900, and (e) dHA-C900 at magnification of 100 k.

Scanning electron microscope observations of the synthesized-HA powders can be seen in Figure 2. High magnification of SEM measurements was taken to locate the particles down to nanometer large surface area. It can be seen that both of cHA-900 and dHA-A900 particles were agglomerated and have irregular round shape. However, the shape of both particles was uniformly distributed. The particles size was calculated using ImageJ software.

The average particle size for both cHA-900 and dHA-A900 was found to be 75.82 nm and 72.48 nm, respectively (*n* = 100). Properties and performance of HA depend on the powder particle size and shape, their distribution, and agglomerates [9]. It is important to achieve nanosized particle as it exhibits many advantages. Nanostructured-HA is said to enhance biocompatibility, bioactivity, and flexibility which provides homogeneous resorption [9, 26]. Many has reported the works on nano-HA; for example, Liu et al. [27] synthesized a needle-like HA with size of 13–170 nm in length and 15–25 nm in width using hydrothermal process. By using wet precipitation technique, Monmaturapoj [28] has successfully synthesized irregular nanosized HA of ~25 nm by varying the concentration of the precursors. However, there was slight difference of HA particles shape, size, and agglomeration with increase of microwave heating. Upon increasing the irradiation time, the agglomeration of particles was less agglomerated but particles from all samples were closely packed. As the time increased, it also can be seen that the particles shape was more uniformly distributed even though their shapes were a bit irregular round shape. As the irradiation time increases, the particle size tended to be larger, that is, 72.48 nm, 75.21 nm, and 76.17 nm for dHA-A900, dHA-B900, and dHA-C900, respectively. It was reported that prolonged microwave irradiation time slowly increased the substance density and decreased the specific surface area. This in turn increased the particles size [29]. Smoleń et al. [29] conducted a synthesis of HA with programmed resorption rate using microwave irradiation as a heating mechanism. All the synthesis conditions such as time, temperature, pressure, and microwave power were computer-controlled. They successfully synthesized nonstoichiometric HA in a very short time of 1.5 min. This almost spherical-like HA dimension was 5.6 nm. As the time increased, the particles size was increased and the particles tended to be less agglomerated with mix structure of spherical and needle-like particles. However, longer irradiation time resulted in increment of Ca/P molar ratio values and stoichiometric HA was obtained at 10 min of irradiation time. There was no change in phase composition and lattice parameters as the time increased. In this study, microwave power showed a significant change in HA morphology. At power of 700 W, the HA particles obtained were almost needle-like shaped with dimension of 38.91 nm in length and 16.43 nm in diameter. Although it was agglomerated, the particles were uniformly distributed. This can be seen in Figure 2(a). By utilizing the eggshells biowaste, Siddharthan et al. [30] reported a fabrication of flower-like HA nanostructure by using microwave heating at 600 W for 10 min. This was done with the aid of ethylenediaminetetraacetic acid (EDTA) which enhanced the crystalline growth. The flower-like nano-HA consisted of leaf-like flakes with dimension of 100–200 nm width and 0.5–1 *μ*m length. However, magnesium from the eggshells was found along with HA produced.

**Figure 3:**
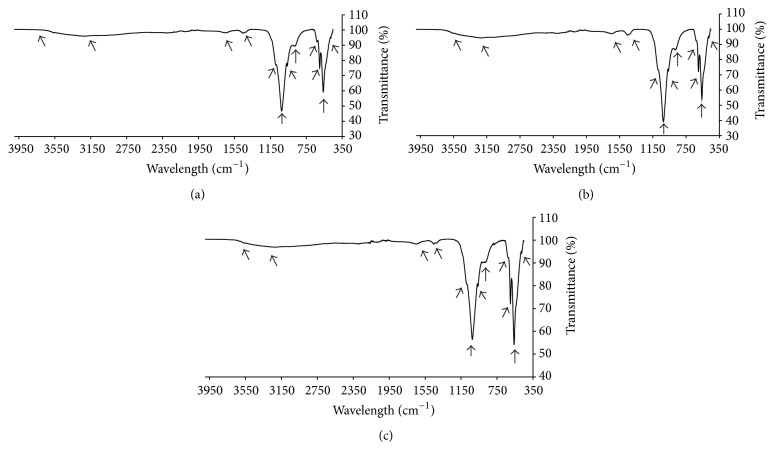
FTIR spectra of (a) dHA-A900, (b) cHA-900, and (c) dHA-700.

The FTIR spectrum of the as-synthesized dHA-A900, cHA-900, and dHA-700 is shown in Figure 3. The FTIR spectrum characteristics of dHA-A900, cHA-900, and dHA-700 are shown in Table 2. All the characteristic frequencies of PO_4_^3−^ modes [31–34] appeared at the frequencies of (I) cHA-900; 472 cm^−1^, 574 cm^−1^, 601 cm^−1^, 962 cm^−1^, 1023 cm^−1^, and 1090 cm^−1^, (II) dHA-A900; 561 cm^−1^, 601 cm^−1^, 962 cm^−1^, 1024 cm^−1^, and 1090 cm^−1^. The stretching vibration of PO_4_^3−^ of V_3_ appeared at 1023 cm^−1^ and 1090 cm^−1^; at 1023 cm^−1^ and 1090 cm^−1^ for cHA-900 and dHA-A900, respectively.

However, the band at 962 cm^−1^ (cHA-900 and dHA-A900) was assigned to V_1_ peaks. The double sharp peaks at 560 cm^−1^ (cHA-900), 561 cm^−1^ (dHA-A900), and 601 cm^−1^ (cHA-900 and dHA-A900) were related to V_4_ of bending modes of P–O bonds in phosphate group [33]. While 475 cm^−1^ (cHA-900 and dHA-A900) was assigned to V_4_ of PO_4_^3−^ modes. These FTIR characteristics of phosphate group confirmed the formation of HA phase. The weak band of liberational mode of structural OH can be seen at 631 cm^−1^ and 629 cm^−1^ for cHA-900 and dHA-A900, respectively. The stretching vibration band of OH (3542 cm^−1^) was invisible due to adsorbed water [25] and the presence of small amount of carbonate at frequencies of ~875 cm^−1^ and ~1450 cm^−1^ which was absorbed from atmosphere during the synthesis process [34]. It is common for biological apatite to allow other nonapatite ions substitution such as carbonate, fluoride, and chloride substitutions which substitutes either OH^−^ or PO_4_^3−^ groups [9, 19]. The broad band at 3214 cm^−1^ (cHA-900) and 3220 cm^−1^ (dHA-A900) was assigned to V_3_ bending mode while peaks at 1645 cm^−1^ (cHA-900) and 1647 cm^−1^ (dHA-A900) corresponded to V_2_ bending mode of adsorbed water molecules. The FTIR spectrum characteristics of HA for sample dHA-700 can be seen in Table 2. As the time increased, all the FTIR spectra occurred and there was no indication of substitution of other functional groups.

## 4. Conclusion

By using both chicken and duck eggshells, irregular circular nanosized HA powders were successfully synthesized with only 15 min of irradiation time with no secondary phases or impurities found. An increase of irradiation time has no effect on the phase composition but there was a slight difference on the particles morphology. By varying the microwave power, various shapes of HA particles can be achieved. The usage of various biowaste materials and microwave heating to fabricate HA has been proven to be low cost, efficient, and fastest technique as the whole process only took about 5 hours from preparation of precursors, mixing of chemical solution, and microwave irradiation to formation of precipitate.

## Figures and Tables

**Table 1:** Sample ID and synthesis condition in the synthesis of HA.

Sample ID	Synthesis condition	
	Microwave power (W)	Irradiation time (min)
cHA-900	900	15
dHA-A900	900	15
dHA-B900	900	30
dHA-C900	900	45
dHA-700	700	15

**Table 2:** FTIR spectra of cHA-900, dHA-900, and dHA-700.

Corresponding assignments	Observed vibrational frequencies (cm^−1^)		
	cHA-900	dHA-900	dHA-700
PO_4_^3−^ bend V_2_	475	475	475
PO_4_^3−^ bend V_4_	560	561	560
PO_4_^3−^ bend V_4_	601	601	601
Structural OH^−^	631	629	631
CO_3_^2−^	877	879	877
PO_4_^3−^ stretch V_1_	962	962	962
PO_4_^3−^ bend V_3_	1023	1024	1023
PO_4_^3−^ bend V_3_	1090	1090	1088
CO_3_^2−^ V_3_	1453	1454	1417
H_2_O adsorbed V_2_	1645	1647	1648
H_2_O adsorbed	3214	3220	3227
Structural OH^−^	3542	3567	3570
